# Neuropsychiatric Manifestations of Arteriovenous Malformation: A Case of Acute Mania

**DOI:** 10.7759/cureus.58297

**Published:** 2024-04-15

**Authors:** Nicole Ann E Villa, Eduardo D Espiridion

**Affiliations:** 1 Psychiatry, Drexel University College of Medicine, Wyomissing, USA; 2 Psychiatry, Drexel University College of Medicine, Philadelphia, USA; 3 Psychiatry, Reading Hospital Tower Health, West Reading, USA

**Keywords:** atypical mania, cerebrovascular disease, arteriovenous malformation, organic mania, acute mania

## Abstract

Current literature shows very few case reports about manic symptoms arising in patients with arteriovenous malformations and no other predisposing factors, where these cases presented with mania before the initiation of treatment. We report a rare case of a 46-year-old male patient, with a history of a left arteriovenous malformation (AVM) status post radiation treatment with associated seizures, who presented to the emergency department of a local hospital with acute mania and other behavioral changes. The patient had manic symptoms, including mood lability, impulsivity, insomnia, decreased appetite, jealous delusions, pressured speech, and suicidal ideations. The patient's escitalopram dose for depression was reduced from 20 mg to 10 mg, and valproate was started during admission. After a three-day hospital admission, his psychiatric symptoms gradually improved. He was subsequently discharged home with additional instructions to follow up with his neurologist. In this case report, we show that organic manic disorder should be considered in any manic patient who presents outside the usual age of onset for idiopathic manic-depressive disease, lacks a family or personal history of affective disturbance, or exhibits concomitant neurologic deficits. In addition, we emphasize that distinguishing between primary psychiatric conditions and those secondary to medical causes for patients who present with acute mania can significantly impact the care a patient receives and can make a difference in their psychiatric and medical prognosis.

## Introduction

Organic mania is a well-described phenomenon that can be attributed to a variety of causes. In patients with acute mania, organic causes must be considered especially when they present atypically, such as a lack of previous psychiatric history or presentation above the age of 40 [1-3]. Mania has been thoroughly researched, with lesions causing mania found to affect regions of the brain that regulate functional control of sleep, appetite, energy, and libido. These areas include the complex interplay between the limbic system, thalamus, and hypothalamus and the frontal lobe, temporal lobe, and basal ganglia [4]. In addition to structural lesions that affect these areas, there are toxic insults that can disrupt the balance of neurotransmitters and chemicals that normally play a vital role in maintaining neurological and psychological homeostasis. Common examples include gamma-aminobutyric acid (GABA) and glutamate neurotransmitters, as well as corticosteroids, antidepressants, and stimulants [4].

There are various neurologic, toxic, and metabolic causes that have been associated with insult to these locations. However, there is only scattered evidence of mania arising secondary to vascular lesions, and more specifically, arteriovenous malformations (AVM). These cases were also present before any of the patients received treatment [5-11]. Thus, we report the case of a 46-year-old male patient with a history of a left arteriovenous malformation (AVM) status post-radiation treatment who presented with acute mania and other behavioral changes.

## Case presentation

The patient is a 46-year-old male, with a past medical history of hypertension, gastroesophageal reflux disease, hyperlipidemia, and spinal fractures after a motor vehicle collision, and was brought into the emergency department (ED) of a local community hospital by the police after being restrained outside the house where his wife and children were staying. According to his wife, he had been banging on the door of this house demanding to see his children, after his wife had left their house a few days ago out of fear for the safety of her children. The wife reported that the patient had been diagnosed with an AVM in the left temporoparietal region one year prior to admission. When the AVM was discovered, he decided to make his wife the decision-maker for his medical care. He subsequently received radiation treatment the same year without any complications. After the treatment, he started exhibiting a variety of symptoms that were concerning for such a drastic departure from his baseline. This included irritability, mood swings, getting upset at his children easily, and holding a gun to his head threatening suicide. The wife also noted he had been paranoid about her infidelity and had been physically abusive to her. He had stretches where he did not sleep for days. This behavior persisted and led to the wife taking the children to a relative’s house, where the most recent situation developed. 

**Figure 1:**
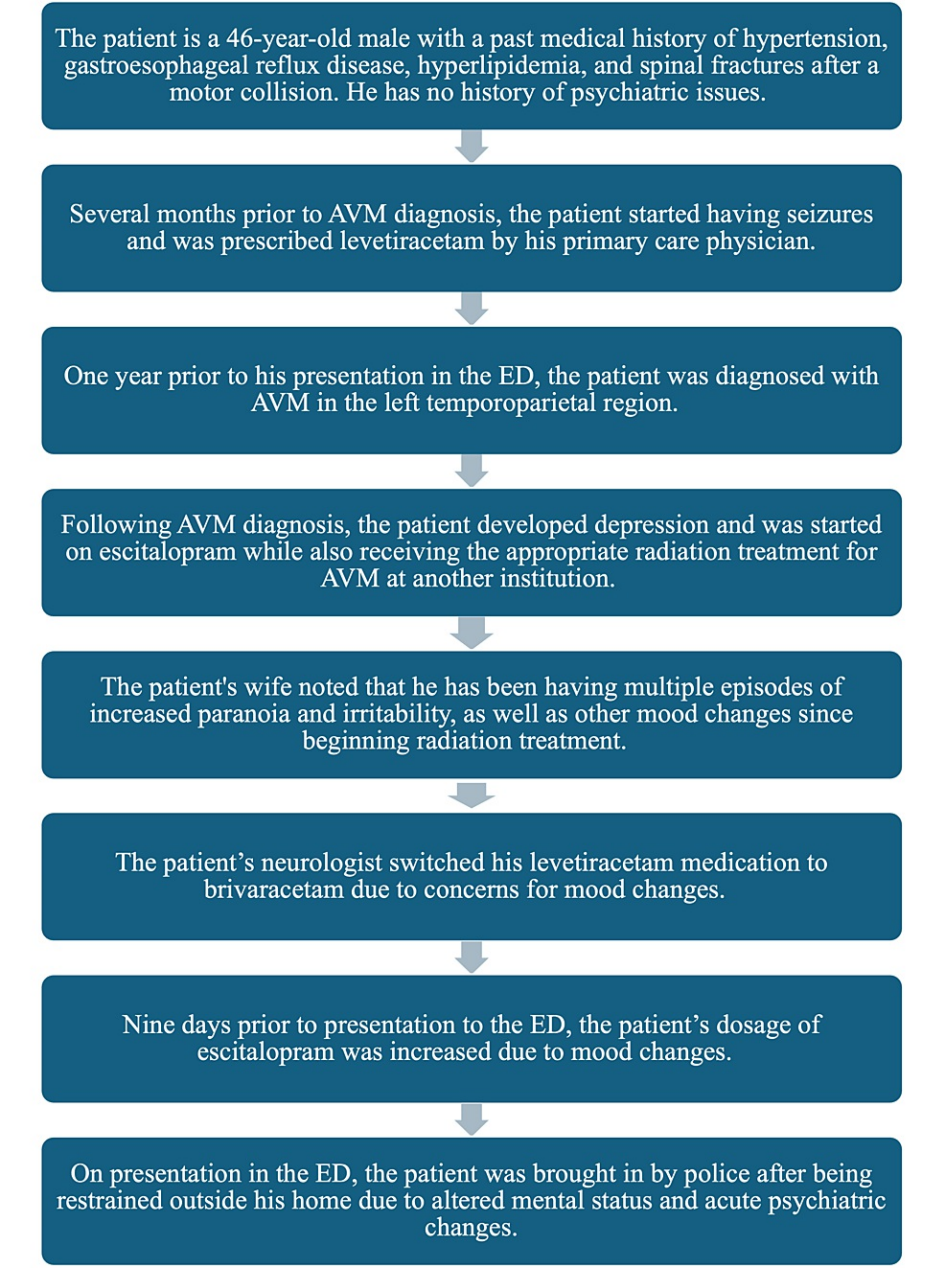
Summary of events prior to presentation at the emergency department

The wife also noted that the patient developed seizures several months before his AVM was diagnosed, for which he started receiving levetiracetam. He had no history of psychiatric illness, but also developed depression due to his AVM and started receiving escitalopram. His levetiracetam was changed to brivaracetam a few months after his treatment due to his neurologists’ concern for possible mood changes due to the levetiracetam. His escitalopram dosage had also been increased from 10 mg to 20 mg nine days before his presentation to the ED. He also had a history of alcohol use of about three to four drinks approximately four to five times a week and cannabis use of hitting a vape pen about twice a week. Events prior to the patient's presentation in the ED are summarized in Figure 1. 

Upon presentation in the ED, the patient's vitals were stable and within normal limits. The neurological physical examination and initial laboratory results were also noted to be within normal limits (Table 1). Further workup consisted of laboratory tests to rule out common causes of secondary mania and psychosis, such as drug use and other metabolic disturbances (Table 2). Serum and urine toxicology screens were only positive for marijuana and were negative for alcohol, salicylate, acetaminophen, and any other recreational drugs. The patient was transferred from the ED to psychiatric emergency services (PES) for further evaluation and imaging. 

**Table 1:** Laboratory results on presentation in the emergency department

Components	Results	Reference
Sodium	142 mmol/L	136-145 mmol/L
Potassium	4.1 mmol/L	3.5-5.1 mmol/L
Blood urea nitrogen	21 mg/dL	7-25 mg/dL
Creatinine	1.01 mg/dL	0.6-1.3 mg/dL
White blood cell	4.9 x 10^3^/uL	4.8-10.8 x 10^3^/uL
Hemoglobin	14.1 g/dL	14.0-17.5 g/dL
Mean corpuscular volume	94.9 fL	80.0-99.0 fL
Platelets	249 x 103/uL	130-400 x 103/uL

**Table 2:** Further laboratory tests performed for workup in the emergency department

Components	Results	Reference
Alkaline phosphatase	36 U/L	33-96 U/L
Albumin	4.5 g/dL	3.4-5.4 g/dL
Aspartate aminotransferase (AST)	20 U/L	14-20 U/L
Alanine aminotransferase (ALT)	27 U/L	10-50 U/L
Total cholesterol	167 mg/dL	0-200 mg/dL
Triglycerides	119 mg/dL	0-150 mg/dL
High-density lipoprotein (HDL) cholesterol	43 mg/dL	35-65 mg/dL
Calculated low-density lipoprotein (LDL)	98 mg/dL	0-100 mg/dL
Valproic acid	50.6 mcg/mL	50-125 mcg/mL

**Figure 2:**
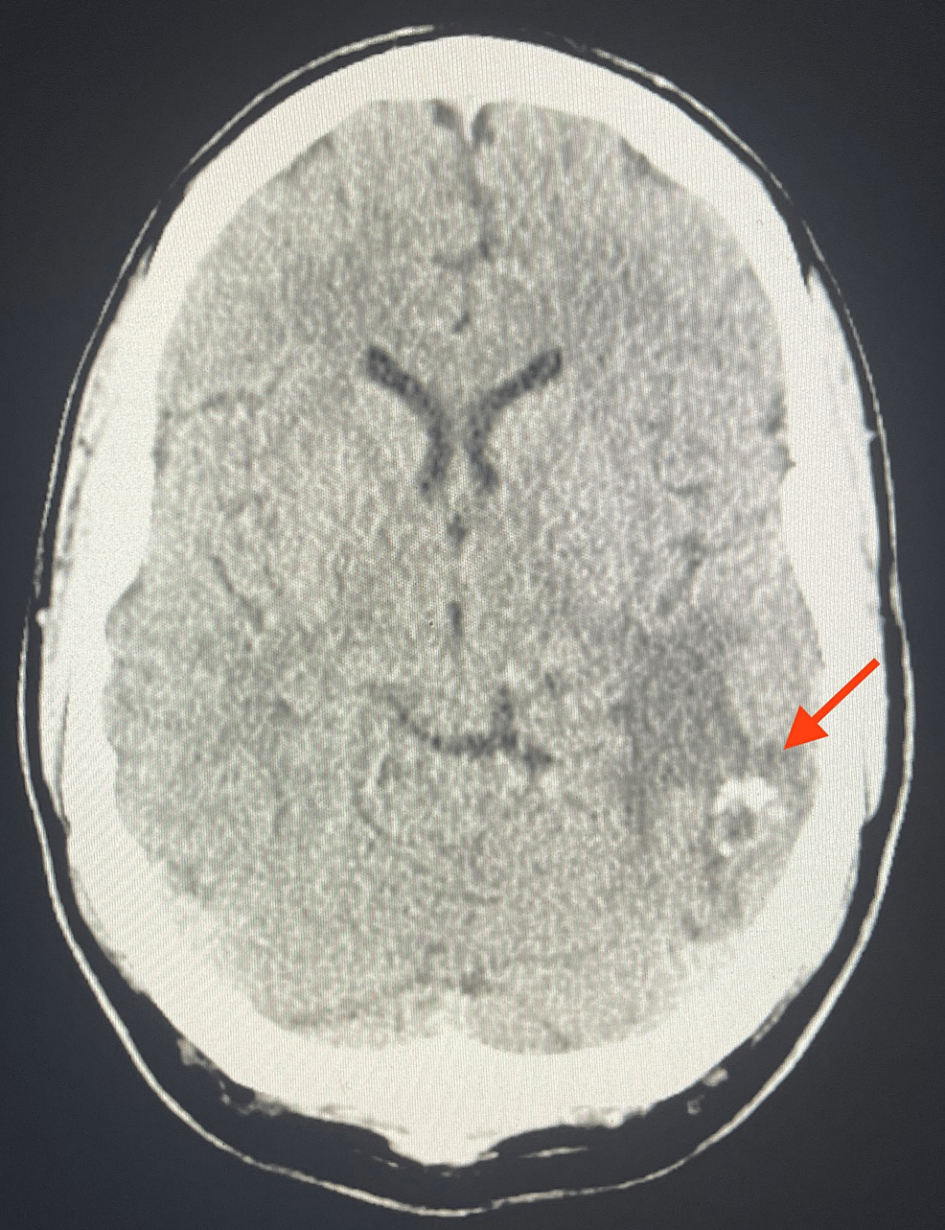
Non-contrast CT performed during admission The non-contrast CT shows a left temporal lobe AVM (red arrow) with no signs of acute infarcts, acute intracranial hemorrhage, and mass.

**Figure 3:**
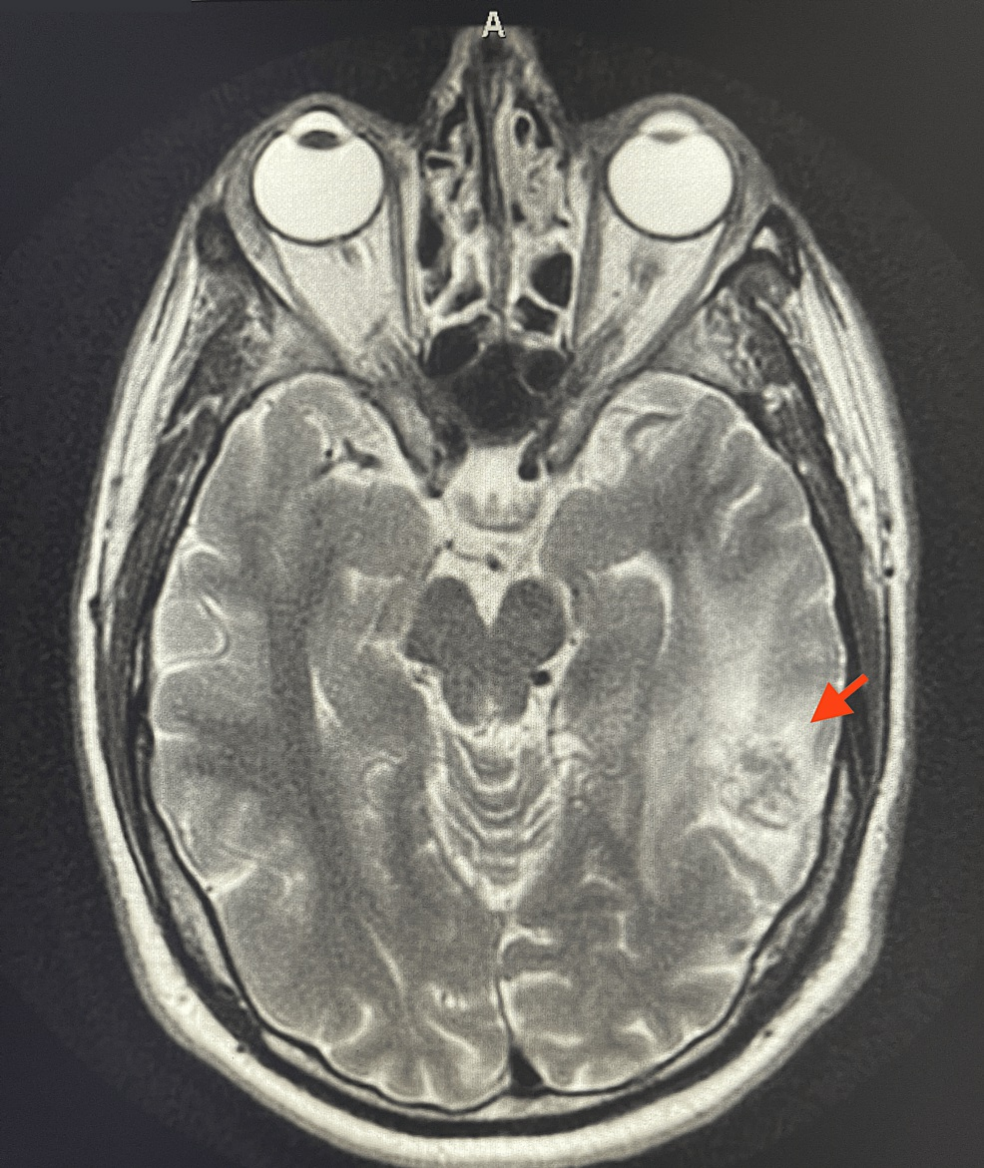
MRI with contrast performed during admission The MRI with contrast shows an AVM status-post radiation treatment in the left temporal lobe, as well as left temporal peri-lesional edema and mass effect of this area (red arrow).

The patient was noted to have had an inpatient stay at another institution six months prior where extensive work-up was performed to assess his AVM post-radiation treatment. Brain imaging was repeated during this admission, and the case was discussed between attendings at both institutions, where it was communicated that the performed non-contrast computed tomography (CT) scan and magnetic resonance imaging (MRI) with contrast were consistent with previous studies of an AVM in remission after radiation treatment. The non-contrast CT showed the known left temporal lobe AVM with no signs of acute infarcts, acute intracranial hemorrhage, or mass (Figure 2). In addition, the MRI with contrast found that the treated AVM in the left temporal lobe was less prominent compared to previous imaging obtained at the other institution. In addition, the left temporal perilesional edema and mass effect were still observed but had similarly decreased (Figure 3).

In the PES, he continued to exhibit extreme mood lability, paranoia, and bouts of agitation. He had multiple spells of crying about his condition and a bleak outlook for the rest of his life, interposed with verbal aggression directed at his wife. The patient remained in PES for three days, and his condition slowly improved over time. He was then transferred to the medical unit, where he was observed to ensure stabilization and to further determine treatment plans. After consulting with neurology, neurosurgery, and medicine, the patient was discharged after a couple of days. His mood had stabilized, and both he and his wife felt comfortable going back home. His escitalopram dosage was reduced back down to 10 mg, and valproate was added for both its anti-epileptic and mood stabilization properties. He was scheduled for outpatient follow-up with his neurologist at another institution.

## Discussion

There have been only a few reported cases of acute mania in patients with an AVM and no prior reported psychiatric history. The most recent one is a 2016 case report describing a 24-year-old male presenting with a headache and manic symptoms [8]. The patient was hospitalized due to his symptoms, and a left-sided AVM was discovered as a part of the workup for the mania. He was treated conservatively with risperidone and divalproex and was eventually tapered off the risperidone without complications [8]. There are also other case reports from 2015, 2011, and 1988, all describing right-sided AVMs found incidentally during the workup for an acute manic episode in a patient [9-11]. In the 1988 report, the patient was a 16-year-old girl who presented similar to this patient, with a seizure disorder and mania resulting in the discovery of an AVM. However, her lesion was surgically resected leading to complete resolution of symptoms [10]. 

In this patient, the presentation varied significantly from the previous case reports, with the most obvious difference being the age of onset. It is likely that this patient was living asymptomatically with the AVM for at least months to years, in which time his brain may have adapted to the altered vasculature to maintain adequate blood flow. In addition, it is well-known that seizures are one of the most common complications of AVMs where his initial presentation was within normal limits [8]. The most important aspect that differs in his presentation is the timing of his mania after the radiation treatment.

Other possible causes for his acute mania include his anti-depressant medication regimen. However, the onset of his manic symptoms did not strongly corelated with the start of his depression nor the start of his anti-depressant medication regimen. In addition, the increase in his escitalopram dosage occurred only nine days prior to admission, with his symptoms having been present and severe before this change occurred. It is possible that this increase in dose may have contributed to the exacerbation that led to the episode and hospitalization, but the cause of the initial onset and persistence of symptoms is not clear [12].

Furthermore, another possible cause for this patient’s presentation is that his temporal-parietal cortex of the brain has become accustomed to the vascular malformation and that, after treatment, the increased blood flow back to the brain may have altered levels of activity in the surrounding regions, which led to the presenting symptoms. This suggests that the radiation treatment shrinking the AVM may have altered long-standing blood flow patterns to his brain and caused varying patterns of hypoactivity and hyperactivity in regions associated with manic symptoms [4]. Supporting this notion, a study from 2018 examined patients with bipolar disorder and the differences in regional cerebral blood flow (rCBF) as compared to healthy patients as a negative control and patients with major depressive disorder as a positive control [1]. It found a significant increase in rCBF to the left temporal lobe and a decrease in rCBF to the right hippocampus. There was also a corresponding increase and decrease in the regional cerebral blood flow velocity (rCBFV) [1]. Another study that examined 23 case reports demonstrated an association between organic mania and disruptions in the networks between the orbitofrontal cortex, dorsolateral prefrontal cortex, and the temporal lobe using functional magnetic resonance imaging (fMRI) [4]. In addition, a case report on a 30-year-old man with bipolar disorder who developed secondary mania 24 years after a head trauma correlates abnormal cerebral blood flow, specifically low blood flow in the orbitofrontal cortex (OFC) and high blood flow in the posterior cingulate cortex (PCC), with acute mania. The dysfunction in these brain regions, along with impaired cognitive functioning may have contributed to the sudden manifestation of mania [13].

Given this patient’s left temporoparietal AVM, history of radiation treatment, and symptomatic timeline, further research into the pathophysiology of organic mania compared to primary mania in bipolar disorder or schizoaffective disorder is warranted.

## Conclusions

This case shows the critical importance of considering medical etiologies of psychiatric presentations, particularly in emergency settings. Distinguishing between primary psychiatric conditions and those secondary to medical causes for patients who present with acute mania can significantly impact the care a patient receives and can make a big difference in their psychiatric and medical prognosis. Furthermore, the complex pathophysiology of arteriovenous malformations and the effects of treatments like radiation highlight the need for further research to understand long-term outcomes and patient prognoses. However, this report also shows the limitation of lacking a validated mania measure, showing the need for comprehensive assessment tools in clinical practice.

Thus, moving forward, clinicians should consider organic etiologies for manic disorders, especially in patients with atypical presentations, to ensure timely diagnosis and appropriate management of acute mania. For instance, organic causes should be considered in any manic patient who presents outside the usual age of onset for idiopathic manic-depressive disease, lacks a family or personal history of affective disturbance, or exhibits concomitant neurologic deficits. Failure to acknowledge these deficits, coupled with mania's tendency to overshadow other clinical aspects, may lead to the oversight of neurologic abnormalities and contribute to the rarity of reported cases of organic manic disorder associated with cerebrovascular diseases.
